# The IIIVmrMLM method uncovers new genetic variants associated with resistance to Fusarium wilt in flax

**DOI:** 10.18699/vjgb-25-41

**Published:** 2025-06

**Authors:** М.A. Duk, A.A. Kanapin, A.A. Samsonova, M.P. Bankin, М.G. Samsonova

**Affiliations:** Peter the Great St. Petersburg Polytechnic University, St. Petersburg, Russia Ioffe Institute of the Russian Academy of Sciences, St. Petersburg, Russia; Peter the Great St. Petersburg Polytechnic University, St. Petersburg, Russia; Peter the Great St. Petersburg Polytechnic University, St. Petersburg, Russia; Peter the Great St. Petersburg Polytechnic University, St. Petersburg, Russia; Peter the Great St. Petersburg Polytechnic University, St. Petersburg, Russia

**Keywords:** flax, Linum usitatissimum, GWAS, Fusarium wilt, Fusarium oxysporum f. sp. lini, лен, Linum usitatissimum, GWAS, фузариозное увядание, Fusarium oxysporum f. sp. lini

## Abstract

Flax (Linum usitatissimum) is an important agricultural crop grown for fiber and oil production, playing a key role in various industries such as production of paints, linoleum, food, clothes and composite materials. Fusarium wilt caused by the fungus Fusarium oxysporum f. sp. lini is a reason of significant economic damage in flax cultivation. The spores of the fungus can persist in the soil for a long time, so obtaining resistant varieties is important. Here we used data on the resistance of 297 flax accessions from the collection of the Federal Center for Bast Crops in Torzhok (Russian Federation) to infection by a highly virulent isolate of the fungus MI39 in 2019–2021. Genotype resistance to infection was assessed by calculating the DSI index, a normalized proportion of genotypes with the same disease symptoms. The IIIVmrMLM program in Single_env mode was used to search for regions of the flax genome associated with resistance. The IIIVmrMLM model was designed to address methodological shortcomings in identifying all types of interactions between alleles, genes and environment, and to unbiasedly estimate their genetic effects. Being a multilocus MLM model, it estimates the effects of all genes as well as the effects of all interactions simultaneously. A total of 111 QTNs were found, of which 34 fell within the body of a known gene or were located in flanking regions within 1,000 bp. The genes into which the detected variants fell were associated with resistance to abiotic and biotic stresses, root, shoot and flower growth and development. Ten of the QTNs found mapped to regions of previously identified QTLs controlling the synthesis of palmitic, oleic, and other fatty acids. QTN Chr1_1706865/Chr1_1706872 and QTN Chr8_22542741 mark regions identified previously in an association search by the GAPIT program. The allelic effect was confirmed for all the QTNs found: a Mann–Whitney test was performed, which confirmed significant differences between the DSI index value in carriers of the reference and alternative allele. An increase in the number of alleles with negative effects in the genotype leads to a statistically significant decrease in the DSI value for all three years of testing. The groups of varieties with a large number of alleles reducing the DSI index had the best resistance. A total of 5 varieties were selected from the collection for which the number of alleles reducing the DSI index value did not exceed the number of alleles with the opposite effect for all three years. These varieties can be used further in breeding programs.

## Abstract (ru)

Лен (Linum usitatissimum) – важная сельскохозяйственная культура, выращиваемая для получения волокна и масла. Лен используют для производства красок, линолеума, в пищевой промышленности, для производства одежды и композитных материалов. Значительный экономический ущерб при выращивании льна наносит фузариозное увядание, вызываемое грибом Fusarium oxysporum f. sp. lini. Споры гриба могут долгое время сохраняться в почве, поэтому получение устойчивых к заражению сортов имеет большое значение Здесь мы использовали данные об устойчивости 297 образцов льна из коллекции Федерального научного центра лубяных культур в Торжке (Россия) к заражению сильнo вирулентным изолятом гриба MI39 в 2019–2021 гг. Устойчивость генотипа к заражению оценивали путем вычисления индекса DSI – нормализованной пропорции генотипов с одинаковыми симптомами болезни. Для поиска районов генома льна, ассоциированных с устойчивостью, использовали программу IIIVmrMLM в режиме Single_env. Модель IIIVmrMLM была разработана для устранения методологических недостатков в выявлении всех типов взаимодействий между аллелями, генами и средой и для несмещенной оценки их генетических эффектов. Поскольку это мультилокусная MLM-модель, она оценивает эффекты всех генов, а также эффекты всех взаимодействий одновременно. Всего было найдено 111 QTN, из которых 34 были локализованы в последовательности известного гена или расположены во фланкирующих районах на расстоянии, не превышающем 1 т. п. н. Гены, в которые попадали обнаруженные варианты, были связаны с устойчивостью к абиотическим и биотическим стрессам, с ростом и развитием корня, побега и цветка. Десять из найденных QTN картировались в областях ранее идентифицированных QTL, контролирующих синтез пальмитиновой, олеиновой и других жирных кислот. QTN Chr1_1706865/ Chr1_1706872 и QTN Chr8_22542741 маркируют районы, идентифицированные нами ранее при поиске ассоциаций программой GAPIT. Для всех найденных QTN был подтвержден аллельный эффект: произведен тест Манна–Уитни, который подтвердил значимые различия между значением DSI у носителей референсного и альтернативного аллеля. Увеличение в генотипе числа аллелей с негативным эффектом приводит к статистически значимому уменьшению величины DSI для всех трех лет тестирования. Группы сортов с большим количеством аллелей, уменьшающих индекс DSI, имели наилучшую устойчивость. Всего из коллекции было выбрано пять сортов, для которых число аллелей, уменьшающих величину DSI, не превышало число аллелей с обратным эффектом по всем трем годам. Эти сорта могут быть использованы в дальнейшем в селекционных программах.

## Introduction

Flax (Linum usitatissimum) is an important crop grown for both fiber and oil. Flaxseed oil is used in the food industry as a source of unsaturated fatty acids and is also used as the main component of varnishes, paints and linoleum. Flax fiber is used in textiles, composites and insulation materials (Goudenhooft et al., 2019). Fusarium wilt caused by the fungus Fusarium oxysporum f.sp. lini limits flax production (Dean et al., 2012). This disease lowers fiber quality and can lead to yield loss in the absence of proactive measures

Primary fungal infection occurs through the roots. The pathogen enters the xylem and blocks the flow of water and nutrients, causing wilting, stem damage and eventually plant death. The spores of the fungus can persist in infested soil for up to 50 years and are very difficult to eliminate (Houston, Knowles, 1949).

Control of Fusarium wilt is possible through various agricultural practices, such as the use of pesticides (Rashid, Kenaschuk, 1993), but the possible harmfulness of pesticides to human health leads to the preference of using varieties resistant to infection, which is an alternative option to control yield loss caused by F. oxysporum (Ondrej, 1993; Rozhmina, Loshakova, 2016).

Resistance to the disease has been acquired through breeding, but the mechanisms of resistance remain incompletely understood. Modern flax varieties have high to medium resistance to Fusarium wilt (Rozhmina, Loshakova, 2016; Rozhmina, 2017). However, co-evolution of pathogen and plant can lead to the emergence of strains with higher aggressiveness or to the loss of resistance in varieties, determined by a small number of genes. Therefore, breeding new varieties with different combinations of genes determining resistance is important for long-term effects. Transcriptomics experiments have shown that cell wall components, transcription factors, secondary metabolites and antioxidants play a prominent role in the response of flax to infection by F. oxysporum f.sp. lini (Galindo-González, Deyholos, 2016; Dmitriev et al., 2017; Boba et al., 2021).

The search for new genomic variants associated with disease resistance and the identification of new genes affecting resistance to fungal infection play a key role in breeding programs. The use of classical GWAS identified QTNs (Quantitative Trait Nucleotides) associated with resistance to Fusarium wilt (Kanapin et al., 2021) and located mainly on chromosome 1, as well as on chromosomes 8 and 13. A large number of QTNs are localized on chromosome 1 within 640 kb (Kanapin et al., 2021; Cloutier et al., 2024).

Plant resistance to disease may also be determined by multiple indirect factors related not only to resistance to fungal infection but also to other plant characteristics, e. g. fatty acids in plants are known to be involved in defense mechanisms against various stressors, including fungal infection (Kachroo et al., 2008; He, Ding, 2020).

Classical MLM-type models help eliminate effects introduced by population structure and sample relatedness, but suffer from the Bonferroni correction for multiple testing, which is too stringent to detect associations with complex traits (Zhang Y.M. et al., 2019). To address this problem, multi-locus MLM models have been proposed that can detect QTNs with marginal effects for which the significance threshold set by the Bonferroni correction is too stringent.

One such method using multi-locus models is the mrMLM method (Zhang Y.-M. et al., 2020) implemented in the IIIVmrMLM package (Li M. et al., 2022). In this paper, we applied this method to search for genomic associations with resistance assessed in infected plants in 2019, 2020 and 2021, which allowed us to identify novel genetic variants not previously detected by classical methods. These variants fell within resistance-related genes as well as within quantitative trait loci (QTL, Quantitative Trait Locus) published previously and related to fatty acid production (You, Cloutier, 2020).

## Materials and methods

297 flax samples from the collection of the Federal Scientific Centre for Bast Crops were grown in Torzhok, Russia. 180 accessions were fiber flax varieties, and 117 belonged to oilseed flax. Of the oilseed samples, 98 belonged to the intermediate type, 4, to large-seeded varieties, and 15, to the crown type.

Resistance of accessions to F. oxysporum f. sp. lini was evaluated under infection-provocation nursery conditions with controlled irrigation but not controlled temperature. Evaluations were conducted in 2019, 2020 and 2021 (Rozhmina et al., 2022). Each variety was replicated 16 times by sowing all seeds in cross rows of containers. The dimensions of the containers were 550 × 85 × 20 cm. Two genotypes, AP5 and I-7, were used as susceptible and resistant genotypes to control Fusarium wilt. The infection background was established by introducing 400 g of pure culture of F. oxysporum f. sp. lini strain MI39. Seeds were planted on the 12th day after inoculation with pure culture of the fungus

Pure culture was prepared by preliminary cultivation of strain MI39 on agar-agar medium with beer wort and subsequent incubation on oat grain substrate (grain/water ratio 1 to 1.75) for 3–4 weeks, until complete infection of oats by the fungus, after which the pathogen was introduced into the soil. The indicator of reliability of the infection background was the reference varieties (resistant and susceptible genotypes), which were sown at the edges and in the middle of each container (16 seeds each). Disease severity was assessed using the Disease Severity score (DSS). The DSS scores ranged from 0 to 3, where 0 was a healthy plant, 1 was a partially blighted plant or stem blight on one side, 2 was a completely blighted plant with seed pods, and 3 was a completely blighted plant that died before pod formation. Based on the DSS, disease severity index (DSI) was calculated using the formula adopted in phytopathology (Guidelines for the Phytopathological Assessment, 2000): DSI = (Σab/3A) × 100 %, where a is the number of plants with the same DSS, b is the DSS score; A is the total number of plants, and 3 is the highest DSS score.

DNA was isolated from leaves using the DNeasy Plant Mini Kit (Qiagen). Whole-genome sequencing of DNA was performed in BGI using the Illumina protocol, which generates paired-end reads of 150 base pairs in length. Comparison with the NCBI ASM22429v2 reference genome assembly (Wang Z. et al., 2012) was performed using bwa-mem (Li H., Durbin, 2009). Variant prediction was performed using NGSEP (Tello et al., 2019) version 4.0; from the 3,416,829 SNPs obtained, 72,526 SNPs were retained after filtering by MAF = 0.05 and conditioning on the presence of the variant in at least 85 % of genotypes. An annotation of the flax genome with the indicated Arabidopsis orthologous genes was provided by the Cloutier group (You, Cloutier, 2020).

Using the IIIVmrMLM package (Li M. et al., 2022) in Single_env mode, GWAS analyses were performed on genetic data filtered by MAF = 0.05. TASSEL (Bradbury et al., 2007) and PLINK (Purcell et al., 2007) with standard settings were used for the necessary data transformation.

The additive effect calculated by the IIIVmrMLM package was used to identify genotypes with high performance. An allele with a negative effect led to a decrease in the DSI in its carriers, while an allele with a positive effect increased the DSI. Varieties were selected in which the number of negativeeffect alleles exceeded the number of positive-effect alleles.

Linkage disequilibrium decay (LD) was estimated using the square of the Pearson correlation coefficient (r2). PopLDdecay version 3.4.1 (Zhang C. et al., 2019) was run to calculate r2 in a 300 kb window. LD decay was calculated based on r2 and distance for each SNP pair using the R script.

## Results

Environmental characteristics may influence disease development. Plants were grown under the infection-provocation nursery conditions with regular irrigation but not controlled temperature. According to the weather station at the growing site, the temperature in the first decade of May was above average in 2019 and 2020 and below average in 2021 (Table 1). In the second decade of May, the temperature was above average in 2019 and 2021 and below average in 2020.

**Table 1.**
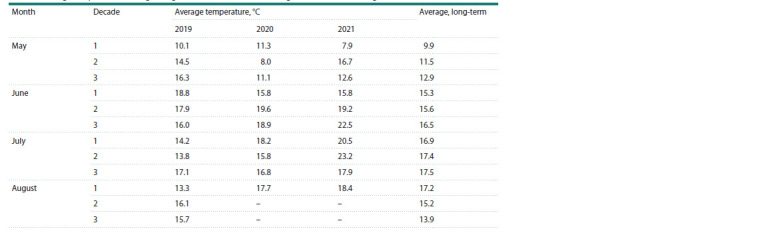
Average temperature of the growing seasons 2019–2021 (according to Torzhok meteorological station)

The analysis of variance showed that the Fusarium wilt infection depends on the year of cultivation and genotype (Table 2). When considering the influence of temperatures, it was found that only the temperature in the 1st decade of May has a significant influence on the variation (F > 1, Pr(>F) < 0.05); moreover, its influence on the Fusarium wilt infection is almost identical to the influence of the year, as can be seen from the values of the root mean square of the residuals in the analysis of variance in Table 2, whereas other environmental characteristics made only a small contribution

**Table 2.**
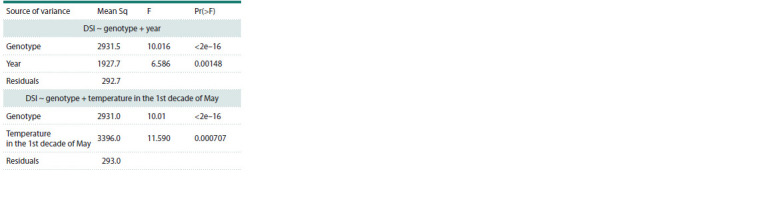
Dispersion analysis

On average, the difference between the maximum and minimum DSI values for genotype in different years is 25.9. Nevertheless, the differences in the DSI for the whole population under consideration from one year to another do not show sufficient significance: when comparing the 2019 and 2020 data, the p-value was 0.996, the 2019 and 2021 data, p = 0.113, the 2020 and 2021 data, p = 0.12.

In other words, despite the large influence of growing conditions, the main interest of the study continues to be the effect of variety (genotype) on disease resistance

**Fig. 1.**
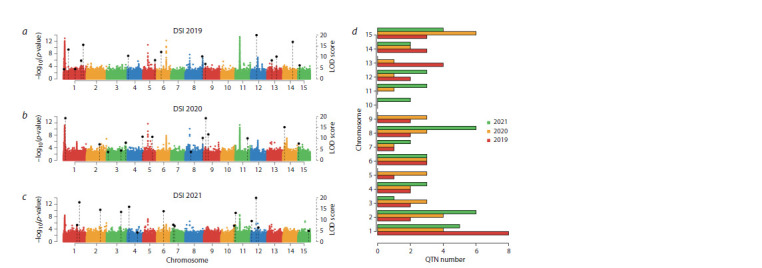
Location of QTNs associated with fusarium wilt relative to chromosomes in flax. a –c – Manhattan plots of GWAS results using the IIIVmrMLM package; black shows QTNs that fell in the QTL or were located near genes along with their LOD score value, which is used in IIIVmrMLM to assess significance; d – distribution of QTNs found for the DSI for the three years data, by chromosome.


GWAS identified 111 QTNs (Supplementary Materials, Table S1)1 associated with the DSI in different years, of which 35 were associated with 2019 data, 37, with 2020 data, and 40, with 2021 data. QTNs associated with data from different years are located on all chromosomes, of which 44 fell within known QTLs (You, Cloutier, 2020; Cloutier et al., 2024) or appeared to be localized in the gene sequence or less than 1,000 bp away from genes (Fig. 1a–c). The distribution of all found QTNs in the genome is shown in Figure 1d. The allelic effect was confirmed for all found QTNs: a Mann–Whitney test was performed, which confirmed significant differences between the DSI value in carriers of the reference and alternative allele (Table S1).

Supplementary Materials are available in the online version of the paper: https://vavilov.elpub.ru/jour/manager/files/Suppl_Duk_Engl.xlsx


The largest number of QTNs found for each year’s infection data were located on chromosomes 1, 2, 8 and 15. In a previous study that used the GAPIT package to find associations with resistance to Fusarium wilt, QTNs were also located on chromosomes 1 and 8 (Kanapin et al., 2021). In total, all the QTNs explain more than 50 % of the variation, at most one QTN explains about 5 % of the variation for one year, as can be seen in Table 3.

**Table 3.**
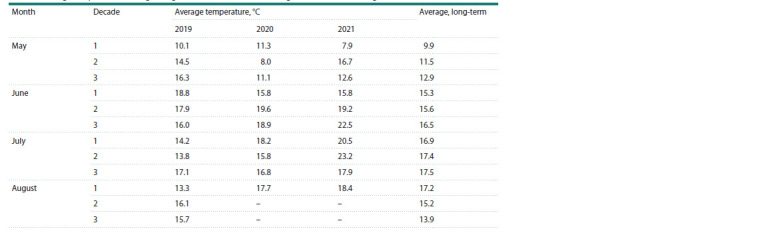
Cumulative percentage of variation in each year’s data explained by QTNs Notе. QTN names are formed as ChrX_N, where X is the chromosome number and N is the position in the chromosome.

Only one QTN was found in the data of two years and three pairs of QTNs found for different years appeared to be located quite close to each other, as shown in Table 4. Table 4 also presents the mean non-normalized DSI values for carriers of the reference and alternative allele for a given QTN. It can be seen that carriers of the alternative allele for all the indicated QTNs showed much lower DSI values than the reference allele carriers. However, of the mentioned QTNs, only one QTN common to the 2020 and 2021 data fell within the sequence of a gene, the function of which, however, is not known, while the other three pairs were located more than 1 kb away from the nearest genes. It can also be noted that QTNs Chr1_1706865/ Chr1_1706872 fell into the previously identified region on chromosome 1, with coordinates 1213418–1854337, associated with resistance to Fusarium wilt (Kanapin et al., 2021).

**Table 4.**
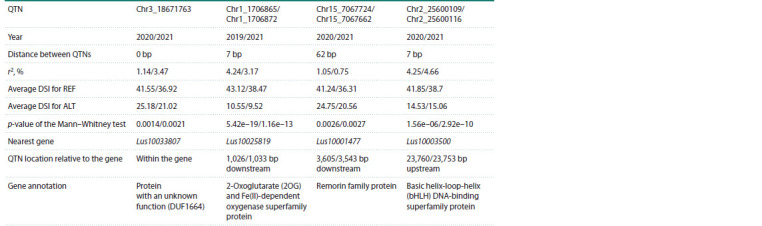
Co-localized QTNs across the years Notе. The corresponding lines show data for different years separated by “/”; bp – base pairs; REF – reference allele; ALT – alternative allele.

Of the 111 QTNs associated with Fusarium wilt resistance in different years, 34 were localized within the gene body or were located at a distance of less than 1 kb from the gene (Table 5).

**Table 5.**
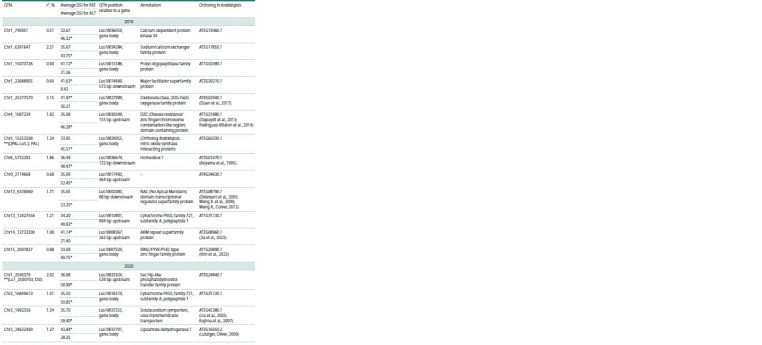
QTNs located within protein-coding genes and their 1-kb flanking regions

**Table 5end.**
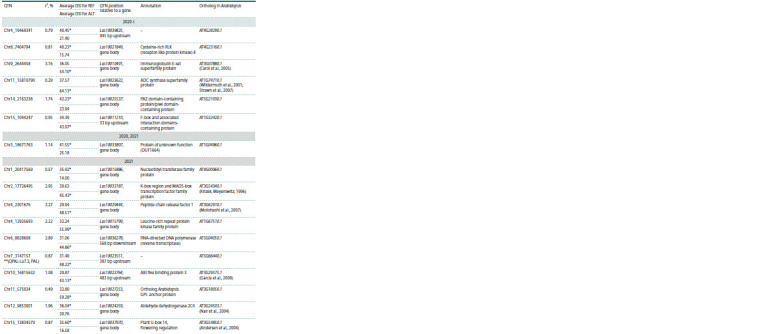
Table 5end. Notе. REF – reference allele; ALT – alternative allele. * The largest of the mean DSI values in carriers of the reference or alternative allele; ** QTNs localized both in the gene body and known QTL.

Within the protein-coding genes and their 1-kb flanking regions, we found 34 QTNs (Table 5), of which 12 had an alternative allele with an effect of decreasing the value of the DSI and 22 with an effect of increasing this value.

10 QTNs fell within the QTLs published previously in (You, Cloutier, 2020), of which two were near a known gene (marked as ** in Table 5). In addition, one QTN fell within a region associated with resistance to Fusarium wilt on chromosome 1 (Table 6), published in (Kanapin et al., 2021; Cloutier et al., 2024).

**Table 6.**
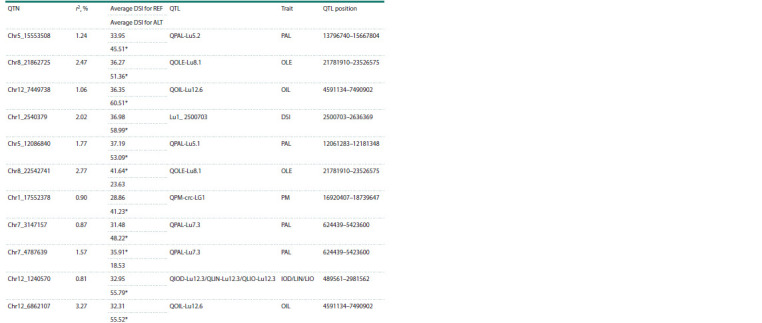
QTNs located within previously identified QTLs Notе. REF – carriers of the reference allele, ALT – carriers of the alternative allele. * The largest of the mean DSI values in carriers of the reference or alternative allele. Abbreviations of trait names from (You, Cloutier, 2020): PAL (Palmitic %) – palmitic acid content; OLE (Oleic %) – oleic acid content; OIL (Oil content %) – oil content, PM (Powdery mildew rating) – powdery mildew rate; IOD (Iodine value) – iodine content; LIN (Linoleic %) – linoleic acid content; LIO (Linolenic %) – linolenic acid content.

Eleven QTNs whose positions overlap with previously identified QTLs from (You, Cloutier, 2020; Cloutier et al., 2024) are shown in Table 6. Most of these QTL are associated with the production of fatty acids: palmitic acid, oleic acid, linolenic acid, etc., and only two QTNs, Chr1_17552378 and Chr1_2540379, fell into QTLs associated with plant immunity. In nine out of eleven cases, the presence of the alternative QTN allele in the plant resulted in an increase in the DSI value, and only in two cases the alternative allele resulted in a decrease in the DSI value compared to the reference allele carriers.

**Fig. 2.**
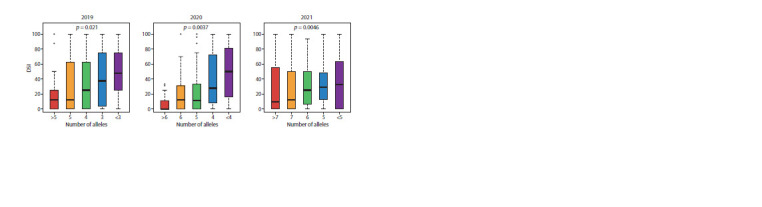
Distribution of DSI values in different years for accessions containing different numbers of alleles that have a negative effect on the DSI value. The upper part of the graphs shows the p-value of the statistical test.

To assess variety performance, the number of alleles with a negative effect (reduction of the DSI value) and with a positive effect was counted among the QTNs found from each year’s data (Table S2). The number of negative and positive alleles affecting the DSI for each year is different, but an increase in the total number of alleles with a negative effect in the varieties leads to a statistically significant decrease in the DSI value for all three years, as can be seen in Figure 2.

Table 7 shows the varieties for which the number of alleles that increased the DSI value did not exceed the number of alleles with the opposite effect in all three years.

**Table 7.**
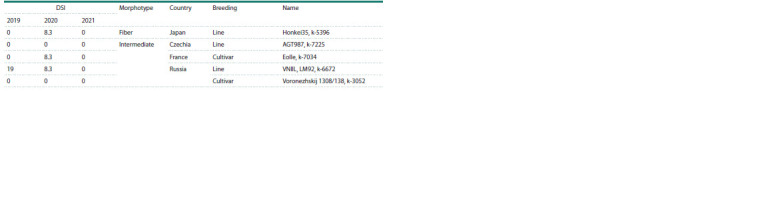
Varieties that had the best combination of alleles with positive and negative effects in all three years

## Discussion

In this paper, we used the IIIVmrMLM program to find genomic regions controlling resistance to Fusarium wilt in flax. A total of 111 QTNs associated with the disease severity index (DSI) value in data from different years were found.

QTNs Chr1_1706865, Chr1_1706872, and Chr8_22542741 apparently point to regions on chromosomes 1 and 8 previously found using the GAPIT package, as published in (Kanapin et al., 2021), being less than the average LD for the corresponding chromosomes, which was 16 and 45 kb for chromosomes 1 and 8, respectively (Fig. S1).

Many of the QTNs fall into or near genes with important functions, and it is possible that these genes are casual (Table 5). Among the QTNs found to fall into genes, there are QTNs that have a favorable effect on a trait. For example, the alternative allele of the QTN Chr1_25377570 in the Lus10027990 gene decreases the DSI which is a favorable effect for this value. The orthologue of this gene in Arabidopsis AT4G02940.1 encodes a dioxygenase that demethylates m6A in mRNA. Mutations in this gene affect the mRNA stability of the flowering time regulators FT, SPL3, and SPL9 and delay the transition from vegetative growth to flowering (Duan et al., 2017). The alternative allele of QTN Chr3_1992356 (Table 5) located in the Lus10037255 gene also increases the DSI value. The Lus10037255 ortholog encodes the urea proton symporter DUR3, which is involved in urea transport across the plasma membrane into root cells (Liu et al., 2003; Kojima et al., 2007). Since F. oxysporum infects plants via roots, the transport of metabolites in roots may influence the susceptibility of the plant to infection.

Some QTNs fall into genes associated with plant immunity (Table 5). For example, QTN Chr15_2097827 with a positive effect (ALT allele increases the DSI) is localized in the Lus10007320 gene, the orthologue of which in Arabidopsis regulates autophagy (Kim et al., 2023). In contrast the alternative alleles of QTNs in the genes Lus10021849, Lus10008367, and Lus10024259 decrease the DSI value. Lus10021849 is an orthologue of Arabidopsis CRK8, which encodes a receptorlike protein kinase. The Arabidopsis orthologue Lus10008367 encodes the effector Ran KA120. This effector prevents autoimmune activation in the absence of pathogens and restricts the activity of the SNC gene, which encodes a TIR-NB-LRRlike receptor involved in the salicylic acid-mediated immune response (Jia et al., 2023). The Lus10024259 orthologue in Arabidopsis is involved in the biosynthesis of ferulic and synapic acids (Nair et al., 2004), which are important for plant resistance to biotic and abiotic stresses

In many cases, the presence of the alternative allele resulted in an increased DSI value in its carriers. Many of the genes that harbored such QTNs were associated with root or leaf growth. For example, Lus10030349 (Table 5) (orthologue AT1G31880.1) encodes the BREVIS RADIX protein, which regulates cell elongation and differentiation in the root and shoot (Depuydt et al., 2013; Rodriguez-Villalon et al., 2014). Lus10036674 (orthologue AT3G01470.1) encodes the HAT5 protein with homeobox and leucine zipper domains that is involved in the mechanism of leaf growth regulation (Aoyama et al., 1995). Lus10010491 (orthologue AT3G07880.1) encodes RhoGDI, an inhibitor of GDP dissociation from Rho GTPase. This inhibitor spatially restricts the sites of growth to a single point on the trichoblast and regulates activation of the RHD2/AtrbohC NADPH oxidase, which is required for root hair growth (Carol et al., 2005).

Mutations in genes related to plant immunity and stress response can also have a negative effect on plant resistance to Fusarium wilt (Table 5). For example, Lus10002083 (orthologue of AT5G08790.1) encodes the ATAF2 protein, which is involved in the regulation of basal defense responses of the host plant against viral infection (Delessert et al., 2005; Wang X. et al., 2009; Wang X., Culver, 2012). Lus10023622 (ortholog AT1G74710.1) encodes chloroplast isochorismate synthase 1, which is involved in the synthesis of salicylic acid, essential for plant defense against pathogens (Wildermuth et al., 2001; Strawn et al., 2007). The AT1G67510.1 orthologue, Lus10015799, encodes an RLK protein kinase rich in leucine repeats. Many RLK kinases are involved in cell response processes to pathogens and abiotic stresses (Lease et al., 1998; Gish, Clark, 2011; Yan et al., 2023). The orthologue of AT3G29575.1, Lus10022764, acts as a negative regulator of abscisic acid (ABA) and stress response (Garcia et al., 2008).

Also, some QTNs are located in genes related to energy metabolism and flower growth. For example, AT3G16950.2, the orthologue of the Lus10037741 gene (Table 5), encodes a dehydrogenase that is a component of the plastid pyruvate dehydrogenase complex (PDC) (Lutziger, Oliver, 2000). This complex is involved in glycolysis. Lus10037741 contains the Chr3_24632490 QTN, in which the alternative allele reduces the DSI value (Table 5). Conversely, the alternative QTN alleles Chr4_2301676 and Chr2_17726495 in the genes Lus10029444 and Lus10033187 increase the DSI value (Table 5). AT3G62910.1, the orthologue of the Lus10029444 gene, encodes the chloroplast peptide chain release factor APG3, which is required for normal chloroplast development (Motohashi et al., 2007). AT3G54340.1, the orthologue of Lus10033187, encodes the homeobox protein APETALA 3, which regulates flower development (Krizek, Meyerowitz, 1996). On the other hand, QTN Chr15_13834579 in the Lus10037970 gene, orthologous to the flowering regulator AT3G54850.1, has a positive effect on resistance to F. oxysporum, reducing the DSI value in carriers of the alternative allele.

It is also interesting to note that 11 of the QTNs found overlapped with previously published functional QTL regions, but only two of these regions were associated with plant immunity, while the rest were related to fatty acid production (Table 6). Fatty acids in plants act as a defense against pathogens and abiotic stresses (Kachroo et al., 2008; He, Ding, 2020); in addition, palmitic acid has been shown to reduce Fusarium infection in other plants (Ma et al., 2021). Thus, QTNs located in regions associated with fatty acid production may influence plant resistance to Fusarium wilt. Four QTNs fell into regions associated with palmitic acid (Table 6), which may indicate an important role of this acid in defense against Fusarium wilt in flax. The Chr8_22542741 QTN overlapped with the QOLELu8.1 QTL associated with oleic acid production, and the Chr8_2256060236 and Chr8_2256060290 QTNs previously found with the GAPIT package (Kanapin et al., 2021) also fell within this region, indicating the possible importance of oleic acid production in protecting the plant against Fusarium wilt. One QTN, Chr1_2540379, also overlapped with a recently published region associated with flax resistance to Fusarium wilt (Cloutier et al., 2024).

**Fig. 3.**
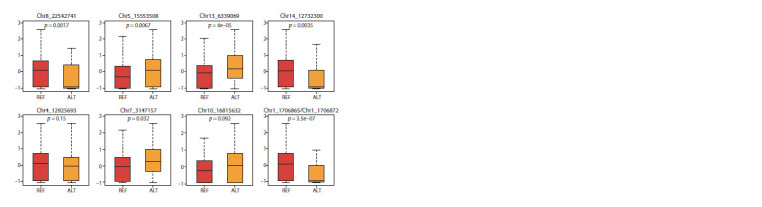
Normalized DSI values in carriers of the reference (REF) and alternative (ALT) alleles in an independent dataset of 100 samples for some QTNs common to both datasets. The p-values of the Mann–Whitney test are shown.

We also tested on an independent dataset of 100 accessions the validity of the detected associations between QTNs and the DSI value (Fig. 3). This dataset grown under the same conditions was previously sequenced separately from the dataset under consideration and does not overlap with the dataset used in this study. It can be noted that the Chr5_15553508 and Chr7_3147157 QTNs, which fell into the palmitic acidrelated regions, and the Chr8_22542741 QTN, which fell into the oleic acid-related region, demonstrate a significant difference in the DSI value between carriers of the reference and alternative alleles in this dataset (Fig. 3). Also, a significant allelic effect is seen in QTNs located in genes involved in plant immunity and stress response (Tables 5 and 6): Chr13_6339069 (Lus10002083), Chr14_12732300 (Lus10008367), Chr4_12925693 (Lus10015799), and Chr10_16815632 (Lus10022764). This suggests that these genes may also be involved in the defense of flax plants against infection.

We identified five varieties with the largest number of alleles decreasing the DSI (Table 7). The DSI of these varieties is much lower than the average DSI value, which for 2019, 2020 and 2021 was 38.7, 38.9 and 34.4, respectively. These varieties can be integrated into modern breeding programs

## Conclusion

As a result of application of the new multilocus model IIIVmrMLM to search for genomic associations with flax resistance to Fusarium wilt wilt, new genomic variants located in important regulatory regions were identified. Varieties with these variants showed greater resistance to the disease and can be used in breeding programs.

## Conflict of interest

The authors declare no conflict of interest.
